# Who Are Happiest?

**Published:** 1882-09

**Authors:** 


**WHO ARE HAPPIEST.**—"Well, Mary, you have had large experience of life; you began early in the families of the poor, and by fidelity to your duties and an ambition to perform them well, you have passed upward, and for years have spent your whole time as monthly nurse in families of wealth, position, and refinement. Now, according to your observation, who are the happiest people?"

"Mechanics' families, ma'am, who are a little fore-handed."

The answer was given with such promptness, and so unhesitatingly, that the mind of the worthy woman must have been made up on mature reflection, and with easy decision.

The answer merits the profound attention of every intelligent parent, and is exceedingly suggestive. The dialogue took place under the circumstances narrated, and without assent or denial, strong reasons may be given for the correctness of the old woman's reply. A lady said to us, just about twenty years ago, that her husband, then deceased, allowed her twenty thousand dollars a year to spend in Paris, while he pulled the political wires at Washington as a senator. "But I was not happy, because politics was an idol before me. I never could be induced to marry a public man again."

The returns of the registrar-general of France show that the middle classes live an average of eleven years longer than day-laborers and the poor.

Our own observation tells us that the sons and daughters of the wealthiest seldom leave heirs to reach maturity, unless those heirs, by reverses, had to begin at the bottom of the ladder, and shove the plane or wield the axe or speed the plow. Mechanics usually begin life poor, and when both husband and wife have a good share of common-sense, they soon unite in their aims, ambitions, industries, and economies, with the result of a gradual increment of their substance. They live in a plain, unostentatious and inexpensive way. The high are so high above them, that they are saved the expense of aping them in style of living, and saved, too, the eating anxieties and cutting mortifications of that most unwise and most unfortunate class of persons who make their whole existence an extended torture, in the weary effort to climb into a sphere in which they have never moved ·

the frequent, frequent cause of the sad wreck of family happiness.

The class above noticed, instead of wasting their attention and their energies in this direction, expend them on the furtherance of their fortune, in the improvement of their pecuniary condition, by curbing immoderate desires. They are not disturbed by any envy toward neighbors who seem to be getting along faster than they are; they derive a quiet happiness in knowing that all they have is paid for; that they have gone nobody's security. Now and then when they see something which would greatly add to their substantial comfort, or would save labor, or protect furniture or clothing, and they have not the means of paying for it, there is a sweetness to them in saving and even in practicing self-denials, until the money is not only earned but in hand, ready to purchase on "the best terms for cash." And the very fact that they have gotten it for less than those who did not pay in hand, gives additional satisfaction; for the difference in price is that much money got without having to work for it. They bring the article home, and talk about its price, and look at it, and turn it over and over again, and appropriate it to its uses with a quiet enjoyment which of itself is worth money; and that is the last of it; while the neighbor who bought on credit, begins, after a short time, to count the days when it is to be paid for, and as the period comes nearer, the uneasiness becomes greater, and with it, actual disquietude. Later on, bills receivable are not met as was expected, then come irritation and anxiety. The children see it; the wife sees it; all know the cause, and peace and happiness and quiet do not dwell in that household; and long before the purchased article is paid for, the pleasure of possession or display has been eaten up, while there is more bitterness in store.

The "fore-handed mechanic," who has the decision to resist the purchase of any coveted article until he has the money to pay for it, finds no trouble, when business reverses come upon a community, in deciding to take in sail while the storm is yet in the distance. He begins to economize, and has got used to it before his neighbors have been able to bring their minds to a decision that it must be done; for few people like to come down, and rather protract the struggle to keep up appearances, in the hope that the times will get better, and they need not make any change. But oh! how wearily the days pass away.

when one is waiting for the hard times to go by, when the meanwhile is spent in painful make-shifts, subterfuges, temporary expedients, and heart-aching sacrifices!

Incomputable are the drawn-out agonies of merchants and bankers and brokers, of clerks, and all salaried persons, in hard times, or even in momentary shocks, which may occur in any week of any year. During these, all domestic happiness, peace, and comfort must be eaten out, and they live a year's suffering in a week. Not so with the "fore-handed mechanic." He bows before the storm of crises with the facility of the reed, and while the angry elements rage above, lies in quiet composure, with the sweet consciousness of perfect safety. There is another element of happiness in our "fore-handed mechanic:" while he and his wife worked into each other's hands, they grew to love each other more in their mutual efforts for bettering their condition. It was a happiness to them to help one another, to save labor and trouble to each other, and their children gradually grew up imbibing the same spirit and temper and feelings; nothing was a trouble to them which in the least saved trouble or money to father and mother; on the contrary, it was a pride and a pleasure and an ambition to save, to help, and to practice self-denial, in the hope of an easier future, which to all was becoming more apparent every day. Hence the happiness!

We see a man every Sunday, who said to his newly-married daughter last year: "My child, go and get you a house for fifteen thousand dollars, and I will furnish it for you." After traversing the city for a month, she said: "Father, I can't find any house that will make us comfortable for less than twenty thousand; can't you get it for us?"

He gave her the title-deed; ordered S—— to put down the carpets, and M—— to supply the furniture; H———— made the china, T—— the silver, M—— the upholstery, and B—— the etceteras of kitchen, pantry, laundry, etc. In short, every thing was procured to her hand, without even the trouble of choosing.

But think you, reader, that this young woman, at the moment of her taking possession of it all, and in any month later, experienced as sweet a satisfaction as does any wife who has helped her husband to earn the money to purchase their first Brussels carpet for their "best room?" Not a bit of it! To get a thing as a gift is pleasant, is gratifying, but to obtain it by mutual individual effort, especially if it has cost some self-denial, is a sweet delight, to which the pampered child of fortune must be forever a stranger. The editor will feel rewarded for writing this, if it shall persuade one subscriber to determine to give each son a good trade; and that each daughter shall feel it her duty to wait upon her mother, to learn to keep house economically, to prepare a sumptuous meal, to spread an appetizing table, to cut and make her own garments, and thus be worthy of a good husband, and be able to help him.

